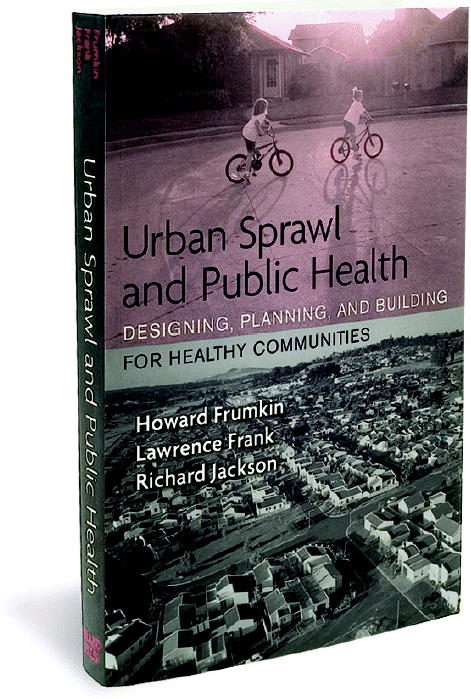# Urban Sprawl and Public Health: Designing, Planning, and Building for Healthy Communities

**Published:** 2005-03

**Authors:** Kenneth Olden

**Affiliations:** Kenneth Olden is the third director of the National Institute of Environmental Health Sciences (NIEHS) and the second director of the National Toxicology Program. He is a cell biologist and biochemist and has been active in cancer research for almost three decades. He was director of the Howard University Cancer Center and professor/chairman of the Department of Oncology at Howard University Medical School, Washington, DC, before coming to NIEHS.

By Howard Frumkin, Lawrence Frank, and Richard Jackson

Washington, DC:Island Press, 2004. 338 pp. ISBN: 1-55963-912-1, $60 cloth.

The press has devoted considerable recent attention to the built environment and its impact on human health and the quality of life. This interest is fueled largely by the obesity epidemic now pervasive in the United States. In fact, the built environment has become a focus for debate about how to control obesity and related health care costs. The National Institute of Environmental Health Sciences defines the built environment as the buildings, spaces, and products created or modified to accommodate modern human society: residential housing, schools, workplaces, parks, roads, walking and biking trails, and commercial centers.

*Urban Sprawl and Public Health*, a timely publication by highly qualified authors, is another wake-up call about public policy decisions with respect to land use, transportation, and community design that adversely affect our health, civic involvement, and quality of life. Its thesis is that the designs of buildings and communities inhibit physical activity and social interactions and promote sedentary lifestyles and environmental pollution or degradation. The authors provide solution-oriented information to professionals, public officials, business and community leaders, and others who are concerned about protecting the environment and human health. They describe sprawl as the way land is used, the way people travel, and the way cities expand over large geographic areas. Such expansion increases distance between places of interest, makes walking and biking impractical, and increases reliance on automobiles for transportation. Although sprawl appears chaotic, the authors point out that it is well orchestrated and supported by public policy through cheap land, favorable tax codes, zoning regulations, lending practices, and the revolution in transportation. The latter made it possible for large numbers of people to live longer distances from work and commercial centers. But now that the forces that spun sprawl (e.g., concerns about sanitation, infectious diseases, noise, crime, industrial pollution, and exposed sewage) no longer represent a nuisance or a threat to safety, it is time to rethink public policies designed to encourage or support migration from urban centers.

Sprawl threatens health in various ways: A sedentary lifestyle increases the risk of cardiovascular disease, stroke, diabetes, and obesity. More than a third of Americans are overweight; the authors note that 35% of people in walkable neighborhoods were found in a recent study to be overweight, compared with 60% in less walkable neighborhoods associated with sprawl. Also, air pollution, another by-product of sprawl, can increase death rates by as much as 26%, primarily from cancer, cardiovascular disease, and pulmonary dysfunctions. Highly polluted air stunts lung growth and development and increases risk for asthma. Sprawl increases automobile injury and death and mental stress and diminishes social capital, the glue that holds families and communities together.

Two solutions proposed by the authors are the development of more mixed-used communities as an alternative to sprawl, and appropriate design of communities to make them more walkable and appealing. However, the authors point out that environmental intervention is not the entire solution to physical inactivity; much is still poorly understood. In fact, sprawl may not be the only, or even the primary, force behind inactivity.

The book is an easy read, enjoyable and informative. The authors were careful to cover both the positive and negative aspects of sprawl. They also emphasized that human health and disease results from complex interactions involving multiple factors. But significantly, they make a compelling case that sprawl is playing an important role.

## Figures and Tables

**Figure:**